# Fabrication of High Impact-Resistant Polyimide Nanocomposites with Outstanding Thermomechanical Properties

**DOI:** 10.3390/polym15224427

**Published:** 2023-11-16

**Authors:** Jimmy Longun, Jude O. Iroh

**Affiliations:** Department of Mechanical and Materials Engineering, University of Cincinnati, Cincinnati, OH 45221, USA; jlongun2008@gmail.com

**Keywords:** optical properties, degree of imidization, polyimide nanocomposite films, polyaniline copolymer/clay nanocomposites, atomic force microscopy, dynamic mechanical analysis

## Abstract

Neat polyimide films are known to be dense and rigid. They are therefore not suitable for use in membranes, sensors and sustainable energy storage applications. In this study, a novel technique has been used to simultaneously improve the porosity, rigidity, damping ability and impact resistance of polyimide membranes. It is demonstrated that dispersion of a small amount of polyaniline copolymer-modified clay of about 0.25–0.5 wt.% into the polyimide matrix resulted in an enhanced storage modulus while maintaining high damping ability and glass transition temperature, T_g_. Novel polyimide/substituted polyaniline-copolymer-clay nanocomposite membranes containing poly(*N*-ethyl-aniline-co-aniline-2-sulfonic-acid)-modified-clay (SPNEAC) was successfully prepared and incorporated into the polyimide matrix to form modified clay/polyimide nanocomposites. UV-Vis analysis of the nanocomposite films shows that the optical transparency of the SPNEAC-PI nanocomposite membranes decreased with increasing SPNEAC concentration due to the high UV-Vis absorption of SPNEAC. Transmittance of about 3% was observed in the nanocomposite membrane containing 5 wt.% modified clay at 500 nm wavelength, which is significantly lower than that for the neat PI membrane of about 36%. The dispersion of SPNEAC containing a high concentration of clay (≥40 wt.% clay), in polyimide matrix, resulted in the attainment of a higher degree of imidization than was possible for the organoclay/polyimide nanocomposite. This behavior is believed to be due to the synergistic interaction between PI and SPNEAC. A correlation of the morphology and elastic modulus of the SPNEAC2/PI nanocomposites shows that at low loading of SPNEAC 2 ≤ 0.5 wt.%, the cross-sectional morphology of the composite is an open, spiky, weblike structure with a storage modulus of about 1 GPa, but it progressively evolves into densely packed microspheroids with storage moduli of ≥2 GPa at 10 wt.% SPNEAC2. The impact energy of SPNEAC/PI composites, calculated from the α-transition peak area, increased with increasing SPNEAC loading and were about 4 times that of neat PI at 10 wt.% SPNEAC.

## 1. Introduction

The need to discover new materials with improved properties is the driving force for many scientific and technological ventures. In the past decade, the area of conducting polymers has attracted enormous attention from many branches of science: physics, chemistry and several engineering disciplines. The desirable electrical, optical, chemical and electrochemical properties of conducting polymers have made their applicability in many technological areas possible [1,2,3,4,5].

Much of the research on conducting polymers, particularly polyaniline, has been focused on understanding its applicability in coatings [6], biosensors [7], light-emitting diodes [8], batteries [9] and microelectronics [10]. However, despite its desirable applicability, polyaniline is insoluble in many organic solvents, such as N-methyl-pyrrolidone (NMP), thereby posing a problem as far as its processing is concerned. In order to circumvent the problem of solubility, previous researchers have explored polyaniline and its derivatives, such as poly(o-anisidine) [11,12,13] and poly(*N*-ethyl-aniline) [14,15,16]. A tremendous research effort has also been dedicated to the study of polyaniline copolymers due to their solubility in common organic solvents such as N-methyl-pyrrolidone [17,18,19,20,21,22,23,24]. Longun et al. [17] synthesized poly(o-anisidine-co-aniline) copolymer by chemical oxidative polymerization and studied its chemical, optical and thermal properties. They reported extended UV-Vis absorption and distinctive chemical features of the copolymer compared to their respective homopolymers.

Previous researchers have studied polyimide/clay nanocomposites and reported increased tensile moduli and thermal stability, as well as decreased permeability to gases [25,26,27,28]. Other recent studies were focused on the use of nanofillers to improve thermal stability [29,30], microwave absorption [31,32,33] and energy storage applications in supercapacitors [34,35,36]. In this study, montmorrilonite clay, Cloisite 30B, was modified by a poly(*N*-ethyl-aniline-co-aniline-2-sulfonic-acid) [SPNEAC] copolymer synthesized by chemical oxidative polymerization. The resultant modified clay was used a filler material in the condensation polymerization of polyimide to determine its effect on the imidization process. Thereafter, the effect of poly(*N*-ethyl-aniline-co-aniline-2-sulfonic-acid)-modified-clay [SPNEAC] on the optical, morphological, chemical, thermal and dynamic mechanical properties of the polyimide nanocomposites was studied. This systematic study is geared towards understanding the properties of the newly created polyimide nanocomposite materials, which combine the desirable optical and electrical properties of polyaniline copolymers and the superior dimensional and thermal stability of clay with the outstanding thermal stability and tensile modulus of polyimide. Incorporation of the polyaniline copolymer/clay filler into the polyimide matrix would also enable the matrix and the copolymer to interact synergistically as a result of their opposite polarity. Polyimide has been shown to be negatively charged [37,38], while polyaniline is positively charged [39], and the interaction between the two polymers can enable the polyimide matrix to play the role of polymeric dopant, thereby influencing the optical properties of the polyaniline copolymer. The suggested intimate interaction between the polyimide and the polyaniline copolymer, in the presence of clay, is also important in regard to the imidization of polyimide, which is thoroughly discussed in this study. It is believed that the intimate interaction between polyimide, clay and the polyaniline copolymer can play an important role in the cyclodehydration process, which is essential for efficient and proper processing of polyimide.

## 2. Materials and Methods

### 2.1. Materials

The reagents used in this study are as follows: aniline-2-sulfonic acid (95% purity), N-ethyl-aniline (98% purity) and N-methyl-pyrrolidone (99% purity), purchased from Sigma-Aldrich Company. Oxalic acid (99% purity), dodecyl-benzene sulfonic acid monohydrate (98% purity), 4,4-oxydianiline (99% purity), pyromellitic dianhydride (99% purity) and ammonium persulfate (99% purity) were also purchased from Sigma-Aldrich Company (St. Louis, MI, USA). Cloisite 30B clay was purchased from Southern Clay Inc, Chicago, IL, USA. The Al-2024-T3 Q-panels were purchased from Q-Labs, Cleveland, OH, USA. All the reagents listed above were of analytical grade; AR. Doubly distilled and de-ionized water were also used.

### 2.2. Synthesis of Poly(N-ethyl-aniline-co-aniline-2-sulfonic acid)-Clay (SPNEAC) Copolymer

Synthesis of Poly(N-ethyl-aniline)-copolymer-clay hybrid was achieved via a single polymerization process. A total of 5.41 g of aniline-2-sulfonic (A2-S), 3.94 of N-ethyl-aniline (NEA), 1.125 of Oxalic acid (OA) and 4.36 of dodecyl-benzene-sulfonic acid monohydrate (DBSA) were added to a beaker containing 150 mL of doubly distilled water, followed by ultrasonication for 3–5 min. Stirring was continued for 30 min using a mechanical stirrer, after which 0.585 g of Cloisite 30B clay was added to the resultant mixture, followed by stirring for 30 min. A total of 4.36 g of ammonium persulfate (APS) was added drop-wise to the mixture, followed by stirring for 24 h. The product was dried in a vacuum oven at 120 °C and then stored in a desiccator.

### 2.3. Synthesis of Poly(amic acid)/Poly(N-ethyl-aniline)-co-aniline-2-sulfonic acid)-Clay(SPNEAC-PAA)

**Figure 1:**
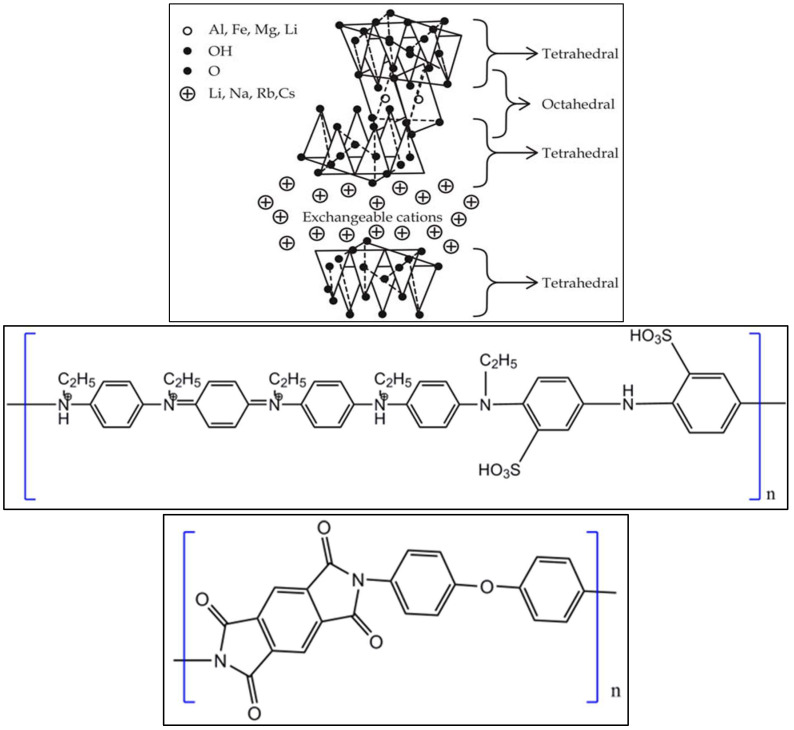
Structure of clay (**top**), substituted polyaniline copolymer (**middle**) and polyimide (**bottom**).

5.1608 g of 4, 4-oxydianiline (ODA) was added to 100 mL of N-methyl-pyrrolidone (NMP) in a round-bottom flask and stirred for 30 min using a mechanical stirrer. Poly(N-ethyl-aniline-co-aniline-2-sulfonic acid)-clay [SPNEAC] (Figure 1) prepared in the previous step was added to the ODA solution, followed by vigorous stirring. After 6 h of stirring, 5.6216 g of pyromellitic dianhydride, PMDA was added to the mixture, and stirring was continued for 15 h. Suspensions were prepared using 0.25, 0.5, 1, 2, 5 and 10 wt.% SPNEAC.

### 2.4. Preparation of Polyimide/Poly(N-ethyl-aniline-co-aniline-2-sulfonic acid)-Clay (SPNEAC-PI) Nanocomposite Films

Polyimide/poly(N-ethyl-aniline-co-aniline-2-sulfonic acid)-clay films were prepared by casting SPNEAC-PAA suspension on glass substrate. Films were prepared by solution casting on glass plate followed by drying in a vacuum oven at 120 °C for 2 h. Final curing was performed in a vacuum oven at 200 °C for 1 h to produce polyimide nanocomposites (Figure 1 bottom) filled with varying amounts of the nanomaterials.

### 2.5. Characterization

Nicolet 6700 FT-IR instrument equipped with Smart Orbit ATR accessory with diamond crystal, from SpectraLab Scientific, Inc, Alexandria, VA, USA, was used to determine the chemical composition of the nanocomposite. ATR analysis was performed over a wavenumber range of 4000 cm^−1^ to 400 cm^−1^. UV-Visible spectroscopy was used to study the optical properties of polyimide and SPNEAC/polyimide nanocomposites. Measurements were performed by using a U-3000 series spectrometer with wavelength range of 190 nm to 900 nm. UV-Vis samples were prepared by dispersing 0.5 mg of the sample in 10 mL of N-methyl-pyrrolidone (NMP). Solid-state UV-Vis was performed by using UV-Vis spectrophotometer. Single cell Peltier accessory was used to measure the transparency of the SPNEAC-PI nanocomposite films. Dynamic mechanical spectroscopy (DMS) was used to study the viscoelastic behavior of the nanocomposites. Measurements were performed from 25 °C to 550 °C using EXSTAR6000, Seiko Instruments, USA Inc., Torrance, CA, USA, under tensile loading mode at a heating rate of 5 °C/min and a frequency of 1 Hz. Thermal transitions were determined by using DSC 6200; Seiko Instruments Inc. tests were performed at a heating rate of 10 °C/min under a nitrogen atmosphere. Cross-sectional morphology of the films was studied using scanning electron microscopy, SEM Hitachi FEG Model (Hitachi, Japan).

SEM samples were prepared by immersion in liquid nitrogen and then fractured using a pair of tweezers to expose the cross-sectional area. A Polaron SC7640 sputter coater was used to sputter coat the samples with silver, Ag.

## 3. Results

### 3.1. Optical Properties

**Figure 2:**
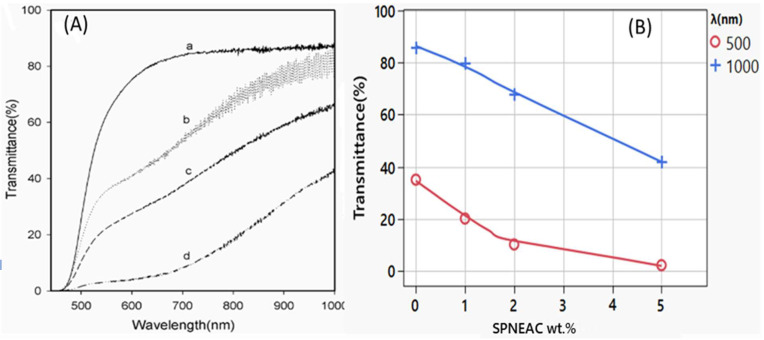
(**A**) Solid state UV-Vis spectra of (a) neat PI and PI containing (b) 1, (c) 2 and (d) 5 wt.% copolymer/clay [SPNEAC]; (**B**) dependence of transmittance on the nanocomposite loading at 500 and 1000 nm wavelength, respectively. Transmittance decreases from 85 for the neat PI to 42% for the nanocomposite containing 5 wt.% SPNEAC in the visible and near-infrared region.

Figure 2A shows the transmittance of PI and SPNEAC-PI nanocomposite films as a function of composition and wavelength. The percent transmittance of the SPNEAC-PI nanocomposite decreases with increasing copolymer/clay (SPNEAC) loading. Percent transmittances of 85, 81, 66 and 42% were obtained in the visible and near-infrared (VIS–NIR) region at 1000 nm for PI nanocomposite membranes containing 0, 1, 2 and 5 wt.% SPNEAC, respectively. The decreased transmittance in the VIS–NIR region is believed to be due to the optical activity of poly(N-ethyl-aniline-co-aniline-2-sulfonic acid) in this region. The effect of the composition of SPNEAC on the optical transmittance at two frequencies (λ), 500 and 1000 nm, is shown in Figure 2B and Table 1. It is shown that a neat PI membrane has the highest transmittance of about 62 and 82% at 500 and 1000 nm, respectively. Interestingly, the optical transmittance for the nanocomposites shows two regimes. The first regime occurs between 450 and 600 nm, and it is due mainly to the matrix (PI), while the second regime, occurring between 700 and 1000 nm, is believed to be due to the contribution from both SPNEAC and the matrix. This observation is in agreement with the data shown in Figure 2, which indicates that the transmittance of SPNEAC-PI decreases sharply with increasing wt.% SPNEAC at frequencies of 500 and 1000 nm (Figure 2).

### 3.2. Functional Group Analysis and Degree of Imidization

**Figure 3:**
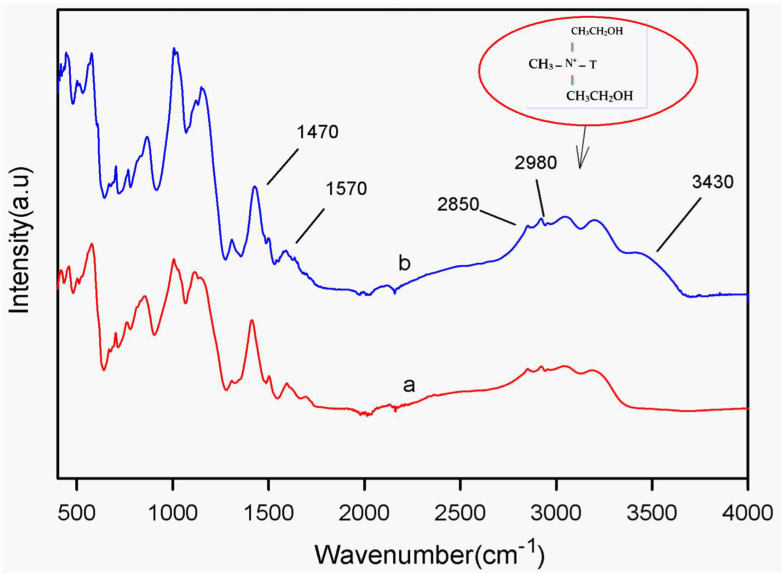
Attenuated total reflectance (ATR) spectra of (a) poly(N-ethyl aniline) (PNEA) and (b) poly(N-ethyl aniline-co-aniline-2-sulfonic acid)-copolymer-clay (SPNEAC) nanocomposite. Characteristic clay peaks are observed in SPNEAC spectrum.

Figure 3a,b show the ATR spectra of the poly(N-ethyl aniline)-copolymer (SPNEA) and the poly(N-ethyl aniline)-copolymer-clay (SPNEAC) nanocomposite, respectively. The ATR spectrum (Figure 3b) of SPNEAC shows an absorption band at 3430 cm^−1^, which is assigned to OH stretching of the surface hydroxyl groups. The absorption bands at 2980 and 2850 cm^−1^ are assigned to the asymmetric and symmetric stretching of the CH_2_ groups in the clay. The absorption bands at 3430, 2980 and 2850 cm^−1^ confirm the presence of copolymer-modified clay. Absorption bands at 1570 and 1470 cm^−1^, due to the quinoid and benzenoid structures, respectively, are shown.

**Figure 4:**
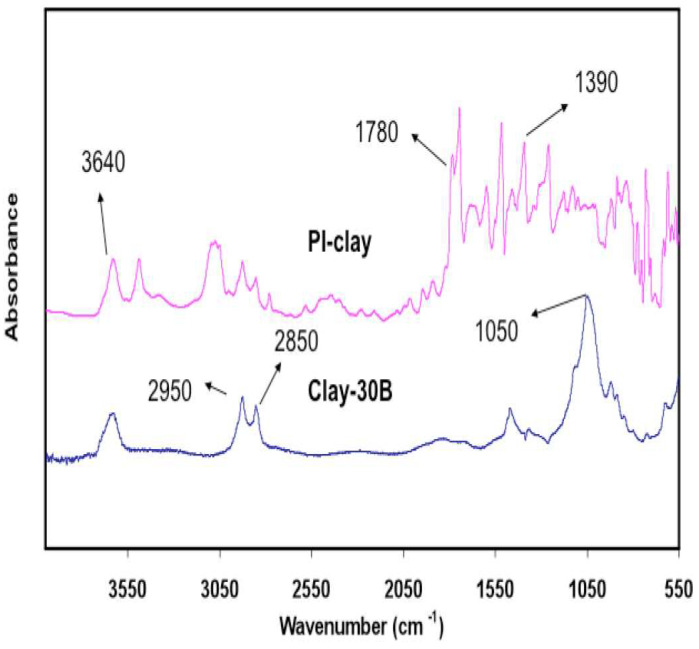
Attenuated total reflectance (ATR) spectra of (**top**) PI–clay nanocomposite, and (**bottom**) Cloisite clay 30B, showing the characteristic absorption peaks for clay and polyimide.

Figure 4 shows the ATR spectra of the 30B clay and PI-30B clay nanocomposites. Typical clay peaks are observed at 2850 and 2950 cm^−1^ due to methylene and methyl groups present in organoclay, and the absorption band at 1360 cm^−1^ is due to C-N stretching of the tertiary amine. The 1470 cm^−1^ absorption band due to the stretching of the phenyl ring is also observed. Figure 3 and Figure 4 confirm the presence of clay in the SPNEAC and PI-clay nanocomposites.

**Figure 5:**
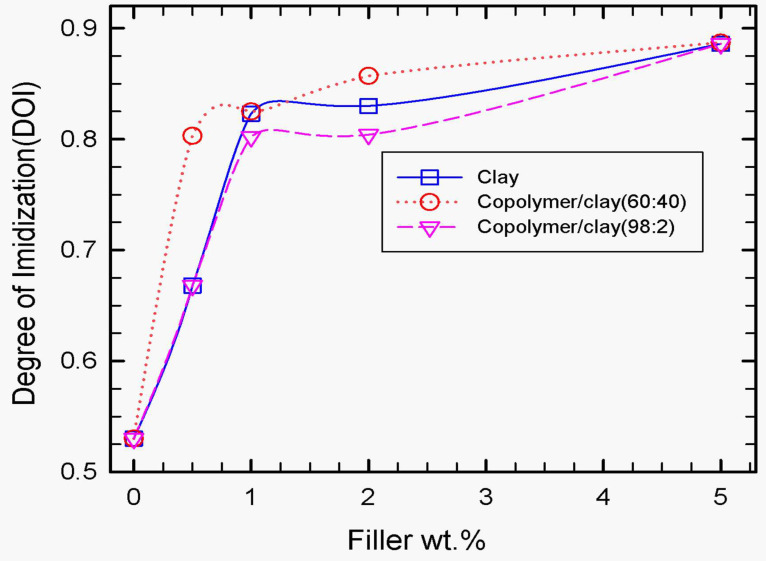
Effect of wt.% organoclay, SPNEAC (60:40) and SPNEAC2 (98:2) on the degree of imidization of PI. The membranes were cured at 150 °C. Both the neat organoclay and copolyaniline-modified clay catalyzed the imidization of polyimide. Addition of about 0.5 wt.% of organoclay, SPNEAC and SPNEAC2, respectively, induced a remarkable increase in the degree of imidization from 53% for neat PI to 68% for neat clay and SPNEAC2/PI and 80% for SPNEAC/PI, respectively.

Figure 5 shows the variation of the degree of imidization (DOI) at 150 °C, with the composition for neat PI and the polyimide nanocomposites membranes. The extent of imidization at 150 °C increased with increasing wt.% nanofiller. Imidization is a very important phenomenon in polyimide and polyimide-based composite processing because it directly affects the thermal and dimensional stability of the resultant material. The degree of imidization (DOI) was calculated using the area of the imide peak at 1778 cm^−1^ with respect to the area of the phenyl peak, 1500 cm^−1^, as shown in Equation (1), where “*A*” denotes the peak area and (*A*_1780_/*A*_1500_)_100%_ refers to the peak area ratio for the 100% imidized polyimide.

(1) $$\alpha =\frac{{\left(A_{1780}/A_{1500}\right)}_{t}}{{\left(A_{1780}/A_{1500}\right)}_{100\%}}$$

The imidization process, which is temperature-dependent, involves the formation of the cyclic imide ring followed by the removal of water. One of the important aspects of imidization is the effect of the curing temperature and clay weight fraction on the degree of imidization (DOI). Tyan et al. [40] studied the effect of clay on the imidization process, and they reported significant enhancement of imidization in the presence of organoclay. Tyan et al. [40] suggested that the enhancement of imidization in the presence of clay is due to the increased surface area and availability of active sites for cyclization and dehydration processes provided by clay. As shown in Figure 5, the degree of imidization (DOI) is sensitive to the concentration of clay and copolymer-modified clay. In the presence of clay, the degree of imidization (DOI), on average, increased from 53.5% at 0 wt.% to 88% at 5 wt.% clay, which represents a 34.5% improvement over the PI matrix. The degree of imidization for the PI-composite membranes containing copolymer/clay (60:40) (SPNEAC) is higher than that of the clay/PI nanocomposites at all concentrations of copolymer/clay. This significant improvement in DOI, which is due in part to the increased polarity and compatibility of the PI matrix and the polyaniline copolymer, is attributed to favorable interaction between the PI matrix, clay and the copolymer during imidization. The addition of the copolymer/clay (98:2) (SPNEAC2) to the poly(amic acid) matrix has a similar effect on imidization as that of 30B clay, especially at a low concentration (≤ 1 wt.%); however, at higher SPNEAC2 loading, the effect on imidization decreases slightly, possibly due to the heterogenous nature of the nanocomposite.

### 3.3. Thermal Transitions

**Figure 6:**
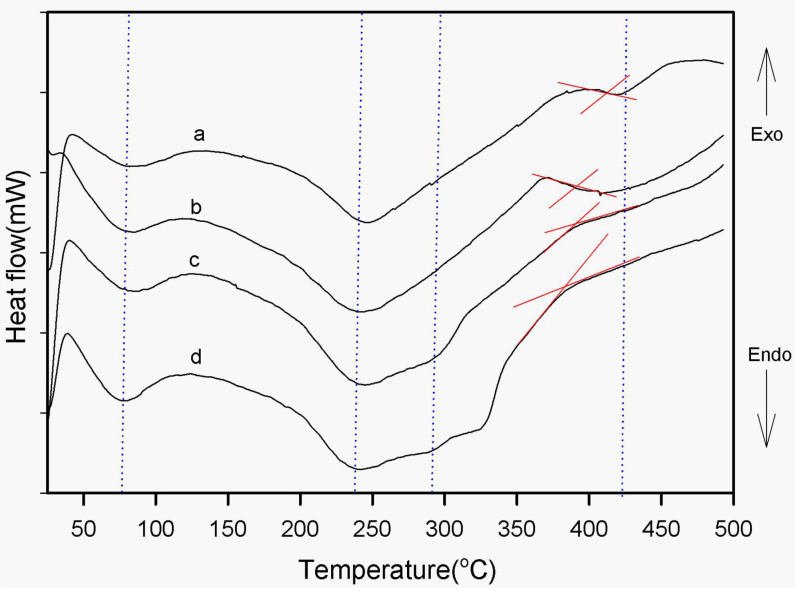
Second trace of DSC thermograms of (a) neat-PI and (b–d) PI nanocomposite membranes containing 0.5, 2 and 5 wt.% copolymer/clay (SPNEAC), respectively. The thermal transitions corresponding to solvent removal, imidization and chain relaxation are shown.

Figure 6 shows the second scan of the DSC thermograms of the neat-PI and the SPNEAC-PI nanocomposite, respectively. The values of the transition temperatures were obtained. The neat PI shows endothermic peaks at ~ 80, 250 and ~415 °C, respectively. The endotherms at 80, 250 and 415 °C are associated with solvent removal, imidization and chain relaxation (glass transition) of polyimide, respectively. SPNEAC-PI also has endothermic peaks in similar regions to the neat PI.

**Figure 7:**
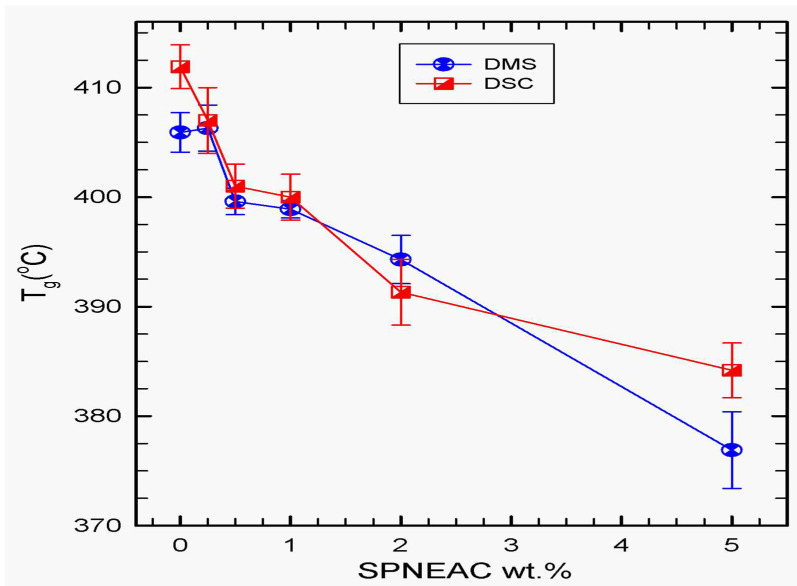
Dependence of the T_g_ of SPNEAC-PI nanocomposites membranes, obtained from DSC and DMS tests, on the wt.% SPNEAC, showing decreasing T_g_ with increasing SPNEAC loading. DMS data shows comparable T_g_ for both PI/SPNEAC containing 0.25 wt.% and the neat PI.

Glass transition often occurs as a step in the DSC thermogram, but, in this case, it is not so distinct. We therefore obtained the T_g_ from the change in slope of the heat capacity and plotted it as a function of composition in Figure 7. The T_g_ obtained using DSC is also compared to that obtained from the dynamic mechanic spectroscopy, DMS, as shown in Figure 7. There is a good agreement in the results obtained from both techniques at low filler loadings of 0.25–1.0 wt.% SPNEAC.

The glass transition temperature of SPNEAC-PI nanocomposites, in general, decreased with increasing SPNEAC wt.%. DMS results shows that, initially, at low wt.% SPNEAC, <0.5 wt.%, a slight increase in T_g_ is obtained over that for neat PI. However, at higher wt.% SPNEAC, ≥0.5 wt.%, the T_g_ of the SPNEAC-PI nanocomposites show a decreasing trend. Comparison of the T_g_ obtained by DSC and DMS shows a similar trend of decreasing T_g_ with increasing SPNEAC wt.%. Better agreement is shown between 0.2 and 0.5 wt.% SPNEAC. Greater disparity in the T_g_ for neat PI and SPNEAC-PI occurred at filler loadings >1 wt.%.

**Figure 8:**
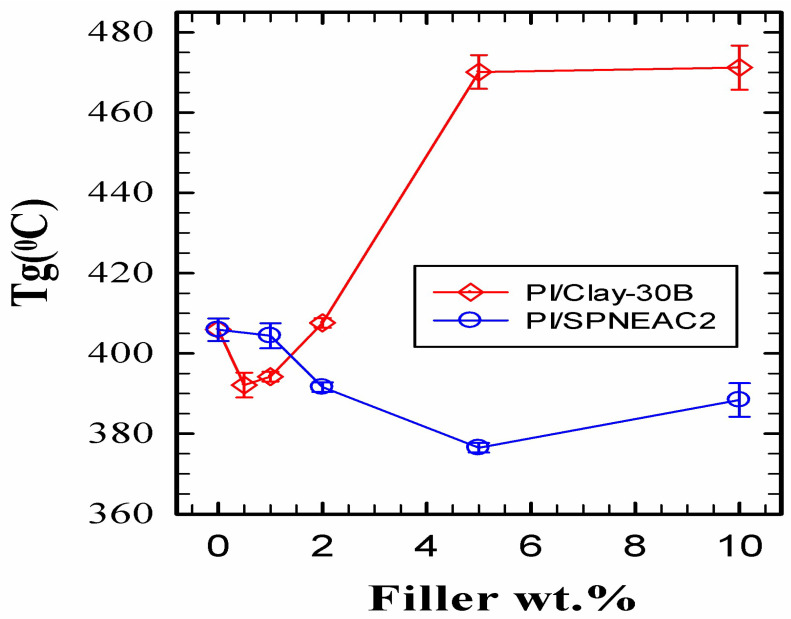
Dependence of the T_g_ of the nanocomposites on the wt.% nanofiller for (top) clay 30B-PI and (bottom) SPNEAC2-PI nanocomposites, measured by DMS operated in tensile mode.

Figure 8 shows a comparison of the variation of T_g_ for clay 30B-PI and SPNEAC2-PI with composition. It shows two distinct regimes of nanocomposite behavior. At low wt.% of nanofillers, <1 wt.%, clay-PI shows a lower T_g_ than the neat PI, while SPNEAC2-PI shows a slightly higher or similar T_g_ than the neat PI. At higher wt.% nanofillers, ≥5 wt.%, the clay 30B-PI nanocomposites show a remarkably higher T_g_ of up to 460 °C. The SPNEAC2-PI nanocomposites, however, show slightly decreasing T_g_ at the higher nanofiller loading of ≥1 wt.%. The differences in the chain relaxation behavior of these nanocomposites can be traced to the chemical structure and morphological differences between the two systems.

### 3.4. Dynamic Mechanical Analysis

#### 3.4.1. Storage Modulus

**Figure 9:**
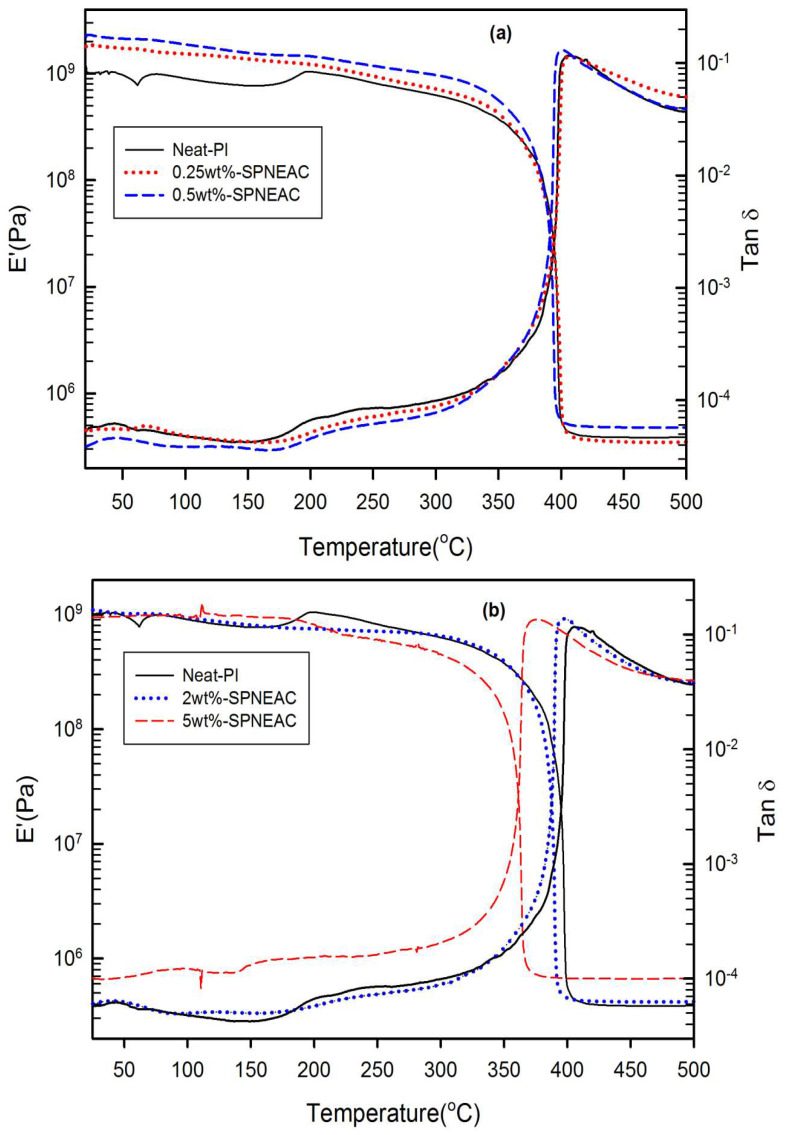
Plot of storage modulus and tan δ against temperature for (**a**) neat PI, and SPNEAC-PI containing 0.25, 0.5 wt.% SPNEAC, (**b**) neat PI and SPNEAC-PI containing 2 and 5 wt.% SPNEAC.

**Figure 10:**
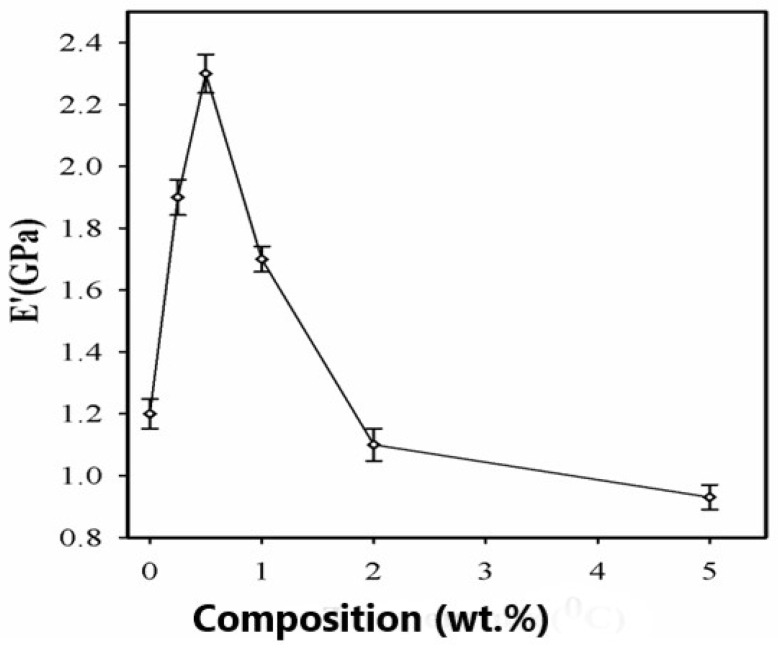
Dependence of the storage modulus of SPNEAC-PI nanocomposites on the wt.% SPNEAC, showing higher T_g_ at low SPNEAC wt.% and, subsequently, a decreasing trend of T_g_ with increasing filler wt.% above 0.5 wt.% SPNEAC.

The storage modulus for SPNEAC-PI nanocomposites is plotted between 25 °C and 500 °C, as shown in Figure 9. The plot of the storage modulus versus SPNEAC loading (Figure 9 and Figure 10) shows a slightly increased modulus with respect to SPNEAC loading between 0.25 and 0.5 wt.%, where a maximum modulus of 2.3 ± 0.025 GPa was obtained. The lowest modulus of the SPNEAC-PI films was 0.93± 0.020 GPa for 5 wt.% SPNEAC. In the rubbery plateau region, there is a sharp decrease in the modulus, and this is often associated with the increased chain flexibility and rubber elasticity. However, the rubbery plateau modulus for the SPNEAC-PI nanocomposite containing 5 wt.% SPNEAC is significantly higher that for the neat PI.

Figure 10 shows the variation of the glassy region storage modulus with SPNEAC-PI composition. At low wt.% SPNEAC between 0.25 and 0.5 wt.%, the nanocomposites show a slightly higher storage modulus of 1.8 and 2.3 GPa, respectively, which is slightly higher than the value of 1.2 GPa obtained for the neat PI. Increasing the SPNEAC loading to 5 wt.% resulted in a slightly decreased glassy region storage modulus and an increased rubbery plateau modulus. The reported increase in the rubbery plateau modulus for SPNEAC-PI containing ≥5 wt.% is due to the presence of physical crosslinks and entangled chains.

#### 3.4.2. Comparison of the Dynamic Mechanical Properties of Clay 30B-PI and SPNEAC2-PI Nanocomposites

**Figure 11:**
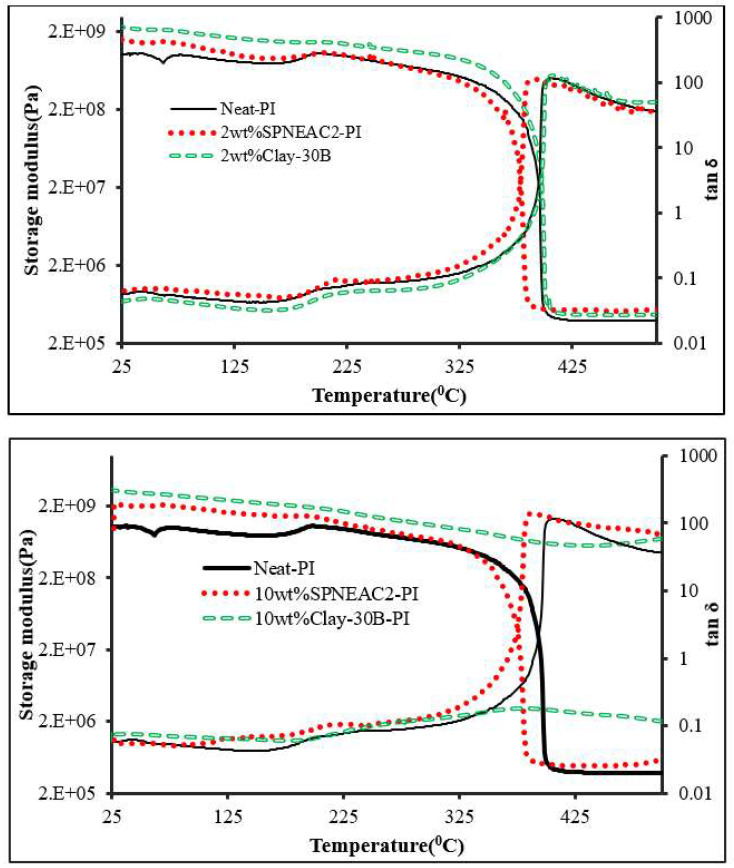
Plot of the storage modulus and tan δ against temperature for (**top**) neat PI, SPNEAC2-PI (2 wt.%) and 30B-PI (2 wt.%) and (**bottom**) neat PI, SPNEAC2-PI (10 wt.%) and 30B-PI (10 wt.%).

**Figure 12:**
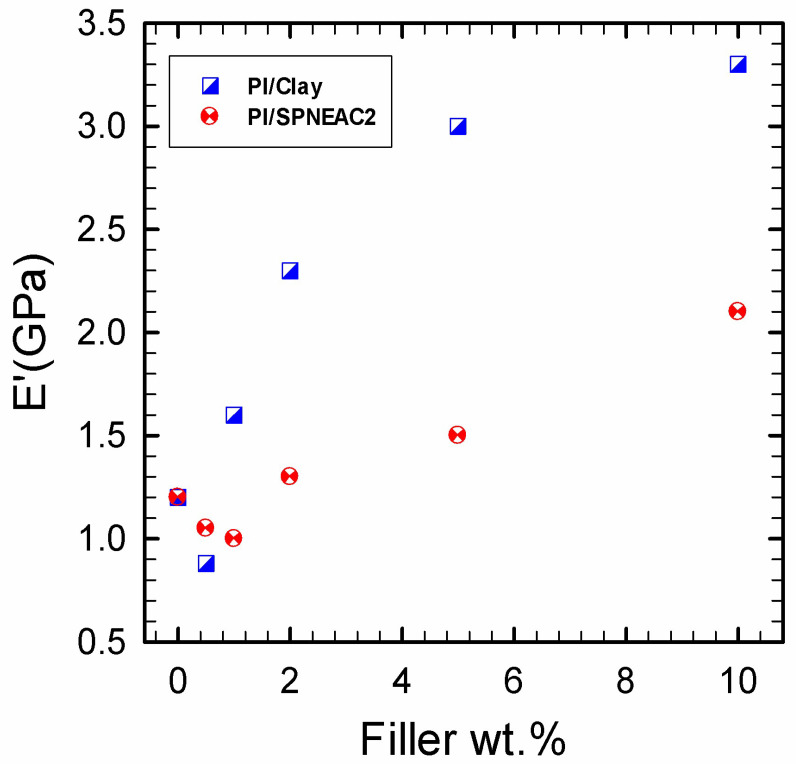
Dependence of the glassy region storage modulus on the weight fraction of (top) neat clay-PI nanocomposite and (bottom) SPNEAC2-PI nanocomposites: Both nanocomposite systems show initial drop of modulus at low filler wt.% ≤1, after which any additional increase in the filler loading resulted in increased nanocomposite storage modulus. It is noted that the neat clay/PI nanocomposites attained higher storage modulus of 3.4 GPa at 10 wt.% clay, which is about 38 wt.% higher than that for the SPNEAC2-PI nanocomposite.

The temperature corresponding to the α-transition peak (Figure 9 and Figure 11) is the glass transition temperature. The glass transition temperature (Figure 9 and Figure 11) generally decreased with increasing SPNEAC weight fraction. The decrease in T_g_ at higher SPNEAC loading could point to increased free-volume and chain flexibility. However, at lower SPNEAC and SPNEAC2 concentrations of 0.25 and 0.5 wt.%, a slight increase in the T_g_ of the nanocomposite over that for the neat PI was observed. It is also noteworthy that the damping ability of the nanocomposite containing 0.25 and 0.5 wt.% SPNEAC and SPNEAC2 is slightly higher than that for the neat PI. Figure 12 shows the variation of the storage modulus for the clay 30B-PI and SPNEAC2-PI nanocomposites with composition. It is shown that increasing wt.% of the nanofillers resulted in an increased storage modulus. Both nanocomposites show a slight decrease in the storage modulus, E’, for nanofiller wt.% below 2 wt.% nanofillers. However, beyond 1 wt.% 30B clay and 2 wt.% SPNEAC2, a remarkable trend of increasing storage modulus with increasing wt.% nanofillers is clearly demonstrated.

#### 3.4.3. Damping Behavior and Impact Energy

Damping is a result of the viscoelastic molecular relaxation phenomenon, which is more pronounced at the glass transition temperature. The height of the tan δ peak (Figure 9 and Figure 11), which is a measure of damping ability, increases with increasing SPNEAC loading, except for 0.25 wt.% SPNEAC-PI. The height of the tan δ peak for α-relaxation increases from 116.5 for the neat PI to a maximum of 137.28 for 2 wt.% SPNEAC-PI. This increase in damping ability at higher SPNEAC loading can be attributed to the open and connected morphology of the nanocomposites, which is believed to impact the relaxation behavior of the nanocomposite. This phenomenon is also associated with the observed decreasing T_g_ values.

**Figure 13:**
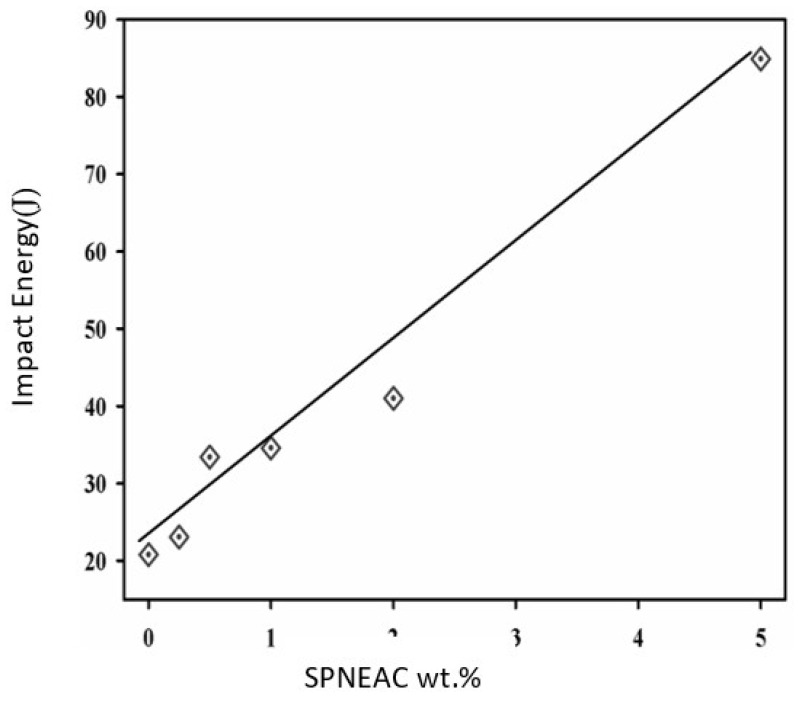
Dependence of the impact energy of SPNEAC-PI nanocomposites on the SPNEAC wt.%.

The area under the tan δ versus temperature curve can be related to the impact energy in accordance with Wada and Kasahara (Equation (2)) [41,42]. The area under the α-transition peak was calculated for the SPNEAC nanocomposites and analyzed as shown in Figure 13. The impact energy of the nanocomposites increased with increasing wt.% SPNEAC. At 10 wt.% SPNEAC, the impact energy of the nanocomposites is about 4 times that for the neat PI.

(2) $$\sigma_{imp}\propto \int_{T_{o}}^{T_{t}}\left(\tan\delta \right)\partial T$$

### 3.5. SPNEAC2-PI Nanocomposite Morphology

**Figure 14:**
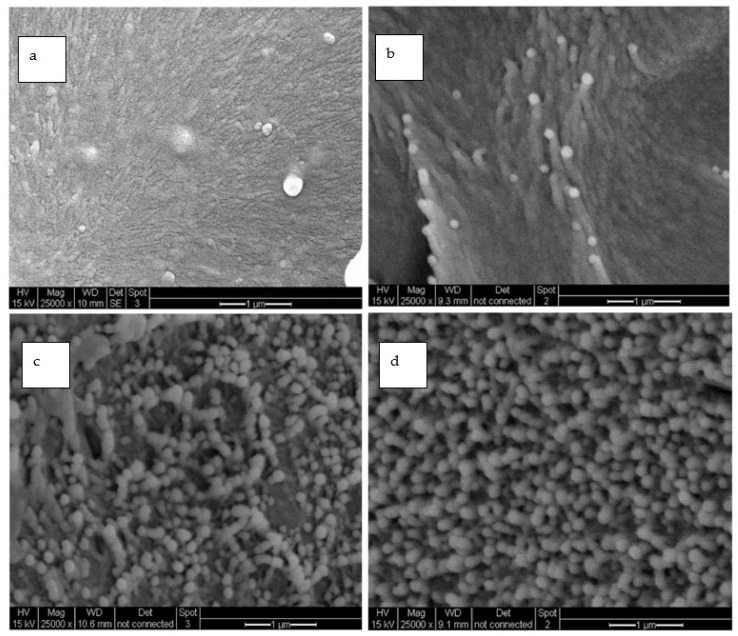
SEM micrographs of (**a**) neat-PI and PI nanocomposite containing (**b**) 0.25 wt.%, (**c**) 0.5 wt.% and (**d**) 5 wt.% SPNEAC2 at 25,000× magnification showing cross-sectional morphology.

Figure 14 shows the SEM images of the neat-PI and SPNEAC2 nanocomposites containing 0.25, 0.5 and 5 wt.% fillers, respectively, at 50,000× magnification. The SPNEAC2-PI nanocomposite film has a dense, fiber-like, columnar morphology at lower loading of SPNEAC2 (≤0.5 wt.%); however, the morphology becomes spheroidal with increasing SPNEAC loading. For the 0.5 wt.% SPNEAC2-PI nanocomposite, discrete and overlapping fibroids are visible throughout the morphology. These features are closely connected, thereby resulting in dense, spiky, columnar morphology. At 2 and 5 wt.% SPNEAC2, the morphology becomes more spherical and connected but uniformly distributed throughout the cross-sectional area. It is suggested that these nanosized particulates contribute to the enhanced storage modulus observed at 5 wt.% SPNEAC2. For the neat-PI films, an amorphous-like diffuse morphology with no distinct features is observed.

**Figure 15:**
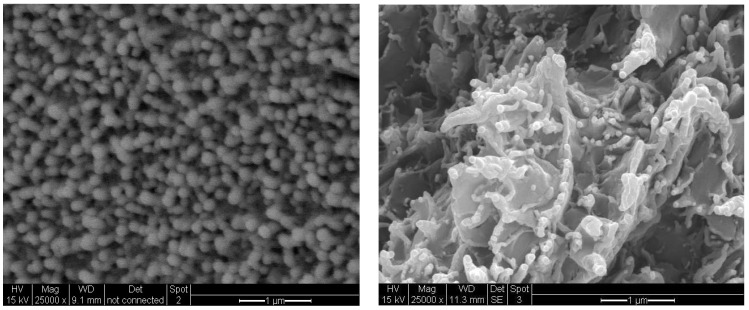
Cross-sectional SEM images of (**left**) SPNEAC2-PI (5 wt.%) and (**right**) 30B clay-PI (10 wt.%) nanocomposites.

The cross-sectional morphologies of 30 B-PI and SPNEAC2-PI containing 10 and 5 wt.%, respectively, are shown in Figure 15. While the SPNEAC2-PI cross-sectional morphology shows close-packed spherical particulate morphology that is a uniformly dispersed spheroid, the cross-section morphology for 30 B-PI shows a dense morphology with spiky edges of 30 B clay sandwiching a smooth, continuous PI matrix (Figure 15).

### 3.6. Discussion of Major Findings

The chemical and electronic structure of the SPNEAC/PI nanocomposites were confirmed by FTIR and UV-Vis spectrophotometry, respectively. FTIR spectra confirmed the presence of both copolyaniline and clay in the nanocomposite, while UV-Vis spectra of the nanocomposite films show that the optical property of the nanocomposite is affected by the optical properties of the constituents. The optical transparency of the SPNEAC-PI nanocomposite decreased with increasing SPNEAC loading because of the high UV-Vis absorption of SPNEAC in the wavelength range between 500 and 700 nm. UV-Vis transmittance of about 3% was observed for the nanocomposites containing 5 wt.% SPNEAC at 500 nm wavelength, while the neat PI had only about 36% transmittance at the same wavelength. SPNEAC/PI containing high concentrations of clay SPNEAC induced a higher degree of imidization than 30B clay, especially at low filler loading of ≤0.5 wt.%. This behavior is attributed to the synergistic interaction between PI and SPNEAC.

A correlation of the morphology and the elastic modulus of the SPNEAC2/PI nanocomposites was made, and it was found that, at low loading of SPNEAC2 ≤ 0.5 wt.%, the cross-sectional morphology of SPNEAC2/PI is a porous, spiky, weblike structure with a storage modulus of about 1 GPa, but it progressively evolves into dense and close-packed nanosized spheroid with a storage modulus ≥ 2 GPa at 10 wt.% SPNEAC2. The impact energy of SPNEAC/PI composites, calculated from the α-transition peak area, increased with increasing SPNEAC loading and was about 4 times that for the neat PI at 10 wt.% SPNEAC.

## 4. Conclusions

Polyimide nanocomposite membranes containing polyaniline copolymer-modified organoclay were successfully synthesized by condensation polymerization. It was observed that the SPNEAC2-PI nanocomposites had nanospheroidal morphology composed of closely packed particles. The addition of copolyaniline/clay nanofillers to PI decreased the transparency of the polyimide membranes mainly due to inherent coloration of the polyaniline copolymer. The reinforcement of PI by Cloisite 30B clay, SPNEAC or SPNEAC2 nanofillers enhanced the degree of imidization (DOI) of the polyimide. The T_g_ of the nanocomposite, obtained from the DSC and DMS analysis, showed a generally decreasing trend with increasing copolymer/clay loading. The observed decrease in T_g_ may be attributed to the less compact morphology of the SPNEAC2-PI nanocomposite membranes observed in the SEM micrographs. The calculated impact energy of the nanocomposites, SPNEAC-PI increased with the increasing weight percent of SPNEAC.

## Figures and Tables

**Table 1:** Effect of wt.% SPNEAC on transmittance.

Composition (wt.%)	Transmittance (%) @ l = 500 nm	Transmittance (%) @ l = 1000 nm
0	36	85
1	20	80
2	12	66
5	3	42

## Data Availability

The data presented in this study are available on request from the corresponding author.
